# Editorial: Exploring processes and applications of metal-microbe interactions

**DOI:** 10.3389/fmicb.2025.1575076

**Published:** 2025-03-04

**Authors:** Eva Pakostova, Carmen Falagan, Alfonso Mazuelos Rojas

**Affiliations:** ^1^MIRARCO Mining Innovation, Sudbury, ON, Canada; ^2^Faculty of Science, Engineering & Architecture, School of Natural Sciences, Laurentian University, Sudbury, ON, Canada; ^3^Faculty of Science and Health, University of Portsmouth, Portsmouth, United Kingdom; ^4^Department of Chemical Engineering, University of Seville, Seville, Spain

**Keywords:** bioleaching, remediation, sulfate reduction, microbiome, metal, pit lake, biofilm, corrosion

Interactions between microorganisms and metals occur in both natural and anthropogenic environments, encompassing a wide range of processes from microbially catalyzed transformations within biogeochemical metal(loid) cycling to microbiology-based applications for metal recovery and removal. The main goal of this Research Topic is to present recent research on metal-microbe interactions and related processes, with the main focus on advances in metal recovery using microorganisms (biomining/bioleaching) and remediation of metal(loid)-contaminated environments. This Research Topic includes 10 manuscripts reporting on metal-microbe interactions, covering bioremediation and biomineralization, cell adhesion, bioleaching, electron transport, and the negative effects of microbes on the quality of industrial fluids.

Microbial processes play a crucial role in biogeochemical cycling, which encompasses mineral formation and weathering, soil and water acidification, carbon capture, nutrient availability, and many other processes. A large number of microbes and their metabolic traits can be harvested to improve industrial applications and processes. Guo et al. reported that an elevated molybdenum content increases the adhesion of *Bacillus subtilis* to the surface of low-alloy steel, and improves subsequent biomineralization that mitigates metal corrosion. The results suggest that molybdenum content can affect the chemotaxis, mobility and carbonic anhydrase secretion-related genes in the bacterium. Guberman-Pfeffer provided a perspective on the electrically conductive filaments of redox-active cytochromes (“nanowires”) in *Geobacter sulfurreducens*, a bacterium that profoundly shapes Earth's biogeochemistry by discharging electrons to minerals and other microbes through the filaments. The above study summarizes our current mechanistic understanding of physiological metal-microbe interactions and thus contributes to efforts to optimize these interactions for bioremediation and energy or chemical production.

There is a growing global interest in implementing “green” biotechnologies in industrial extractive processes, and bioleaching is a cost-effective alternative to conventional pyro- and hydrometallurgical processing that has reduced negative environmental effects. Lithium is becoming increasingly important due to its use in batteries needed for electrification and the transition to net zero. Kirk et al. investigated the bioleaching of lithium from three different minerals using the acidophile *Acidithiobacillus ferrooxidans*, including performing kinetic modeling to predict the dominant reaction pathways for lithium extraction. The study successfully demonstrated the potential for acidophilic bioleaching in jadarite processing, with implications for other lithium-bearing deposits. Antimony (Sb) is another important strategic material, mainly used as a flame retardant in electronic devices, batteries, printing industries, semiconductors, and pharmaceuticals. Zheng et al. improved the dissolution rate of stibnite (Sb_2_S_3_) by adding pyrite (FeS_2_), which led to the formation of FeS_2_-Sb_2_S_3_ galvanic cell and promoted the electron transfer efficiency and antimony extraction by *Sulfobacillus thermosulfidooxidans*. Importantly, the mechanism of (bio)leaching of Sb-bearing sulfides and chemical speciation was described based on the results obtained via a combination of experiments and modeling.

Metal mining generates large amounts of waste (such as waste rock and mine tailings) that are stored at or near mine sites and present serious risks to the environment due to acid mine drainage (AMD) and the mobilization of hazardous metal(loid)s that cause problems for humans and wildlife. A wide range of remediation strategies have been developed to mitigate the negative environmental impacts of mining, and many of these strategies are based on and affected by microbial activity. Sulfate-reducing bacteria (SRB) are often used in both active and passive mine water treatment to immobilize metal(loid) contaminants as insoluble sulfides. The majority of known SRBs are neutrophils, but species that are capable of sulfate reduction under acidic conditions are of particular interest for practical applications due to many mine-impacted environments being acid-generating. The metabolic activity of acid-tolerant SRBs was enhanced in the deep layer of an acidic pit lake in Spain, via sulfur and organic amendments that promoted the formation of low-solubility sulfide minerals (Liu et al.). Microbial community analysis of enrichment samples revealed the dominance of *Desulfosporosinus acididurans*. Dong et al. applied SRBs to treat synthetic AMD and real mine tailings from a zinc-lead mine, reporting on the metal tolerance and bio-cementing strength of SRBs. The mechanism by which SRBs fix pollutants in tailings was revealed via a detailed analysis of the solids.

Revegetation is an environmentally sustainable technique for *in situ* mine site remediation, and it is less costly than traditional physicochemical techniques. However, due to the elevated levels of dissolved metal(loid)s and low microbial activity in the tailings area, it is generally difficult for plants to survive. Mao et al. studied the specific microbiome associated with *Imperata cylindrica*, a dominant pioneer plant in many abandoned mines. The authors reported an increased diversity of fungi in the rhizosphere soil, which is expected to lead to enhanced nutrient availability and thus improved mine restoration efficacy. In recent years, bacterial exopolysaccharides (EPS) have been investigated as an emerging approach in the remediation of mine-impacted environments contaminated with heavy metals. A genomic study by Najjari et al. evaluated the metal biosorption potential of EPS produced by a novel *Psychrobacillus* strain isolated from an iron ore deposit in northern Tunisia, indicating greater adsorption of iron and lead compared to copper and cadmium. Another innovative approach, electrokinetic remediation, is based on metal removal by low-potential electrodes installed into contaminated soil. Narenkumar et al. investigated the potential of biosurfactants produced by *Pseudomonas stutzeri* and *Bacillus cereus* to serve as electrolytes during the electrokinetic process, achieving the removal rate of 70–75% of chromium from contaminated soil, without negative effects on plant seed germination.

In addition to positive effects, microbial activity can negatively affect material quality, and promote deterioration and corrosion. It has been shown that microbial proliferation in metal-cutting fluid decreases its quality; organic acids secreted by anaerobic microorganisms and decomposition of some of the fluid components by aerobes lowered the pH of the cutting fluid and its corrosion resistance. In addition, the accumulation of fungal mycelium resulted in reduced lubricity and poor stability (Shen et al.).

In summary, understanding microbial processes and metal-microbe interactions can help develop effective measures to promote beneficial microbial processes (such as metal extraction during bioleaching, metal immobilization in remediation systems, and formation of protective layers on metal surfaces) and inhibit deleterious microbial activities (e.g., deterioration of industrial materials).

